# Supplementary material for: Automated Approach to In Vitro Image-Guided Photothermal Therapy with Top-Down and Bottom-Up-Synthesized Graphene Quantum Dots
Source: Nanomaterials (Basel). 2023 Feb 22;13(5):805. doi: 10.3390/nano13050805 (PMC10005083; doi:10.3390/nano13050805)
Supplement: Supplementary file 1 [file nanomaterials-13-00805-s001.zip › nanomaterials-2220283-Supplementary.pdf]

Automated Approach to *In Vitro* Image-Guided Photothermal Therapy with Top-Down and Bottom-Up-Synthesized Graphene Quantum Dots

Bong Lee[1], Gretel A. Stokes[1], Alina Valimukhametova[1], Steven Nguyen[1], Roberto Gonzalez-Rodriguez[2], Adam Bhaloo[1], Jeffery Coffer[3], Anton V. Naumov[1]*

[1]Department of Physics and Astronomy, Texas Christian University, Fort Worth, TX
[2]Department of Physics University of North Texas, Denton, TX
[3]Department of Chemistry and Biochemistry, Texas Christian University, Fort Worth, TX
*Correspondence: a.naumov@tcu.edu; Tel.: +1 (713) 253-8775

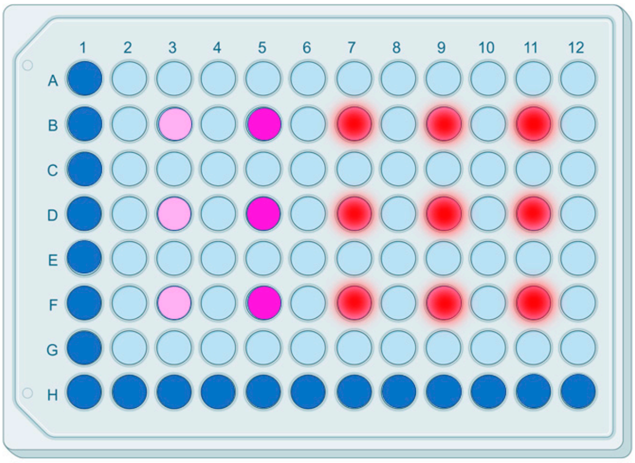

Figure S1. *In vitro* PTT setup for a 96-well plate: HeLa cells control (pink), GQD-treated cells (purple), GQD-treated and laser-irradiated cells (red), and heat sink/cleaning station wells for the thermocouple (blue).

Table S1. The code controlling the 3D printer for *in vitro* PTT experiments written using computer numerical control program language G-code.

Code Block	Purpose
**;FLAVOR:Marlin<br>;TIME:0<br>;Filament used: 0m<br>;Layer height: 0.2<br>;MINX:2.14748e+006<br>;MINY:2.14748e+006<br>;MINZ:2.14748e+006<br>;MAXX:-2.14748e+006<br>;MAXY:-2.14748e+006<br>;MAXZ:-2.14748e+006<br>;Generated with Cura_SteamEngine 4.6.1**	**Start Program**

**M140 S44; Set platform temperature at 44 °C<br>M105; Report temperature<br>G28; Home all axes<br>G0 X0 Y0 Z25 F10000**	Warm up platform and position the thermocouple.
**G4 S300**	Standby for 10 min to warm up wells solution to 37 °C.
**G0 X101.4 Y149 Z25 F10000<br>G0 X101.4 Y149 Z7 F10000; Dip in<br>G4 S1<br>G0 X101.4 Y149 Z25 F10000; Dip out<br>G0 X101.4 Y149 Z25 F10000<br>G0 X101.4 Y149 Z7 F10000; Dip in<br>G4 S1<br>G0 X101.4 Y149 Z25 F10000; Dip out<br>G0 X101.4 Y149 Z25 F10000<br>G0 X101.4 Y149 Z7 F10000; Dip in<br>G4 S1<br>G0 X101.4 Y149 Z25 F10000; Dip out**	Thermocouple cleanup.
**G0 X154 Y148 Z25 F10000; Laser Start Position<br>G4 S30<br>G0 X119.4 Y139 Z25 F10000<br>G0 X119.4 Y139 Z7 F10000; Dip in<br>G4 S1<br>G0 X119.4 Y139 Z25 F10000; Dip out<br>G0 X119.4 Y139 Z25 F10000<br>G0 X119.4 Y139 Z7 F10000; Dip in<br>G4 S1<br>G0 X119.4 Y139 Z25 F10000; Dip out<br>G0 X119.4 Y139 Z25 F10000<br>G0 X119.4 Y139 Z7 F10000; Dip in<br>G4 S30<br>G0 X119.4 Y139 Z25 F10000; Dip out<br>G0 X101.4 Y140 Z25 F10000<br>G0 X101.4 Y140 Z7 F10000; Dip in<br>G4 S1<br>G0 X101.4 Y140 Z25 F10000; Dip out<br>G0 X101.4 Y140 Z25 F10000<br>G0 X101.4 Y140 Z7 F10000; Dip in<br>G4 S1<br>G0 X101.4 Y140 Z25 F10000; Dip out<br>G0 X101.4 Y140 Z25 F10000<br>G0 X101.4 Y140 Z7 F10000;Dip in<br>G4 S1<br>G0 X101.4 Y140 Z25 F10000;Dip out**	Temperature measurement at well B3; followed by thermocouple cleanup.

**G0 X172 Y148 Z25 F10000;Laser Start Position**<br>**G4 S30**<br>**G0 X137.4 Y139 Z25 F10000**<br>**G0 X137.4 Y139 Z7 F10000;Dip in**<br>**G4 S1**<br>**G0 X137.4 Y139 Z25 F10000;Dip out**<br>**G0 X137.4 Y139 Z25 F10000**<br>**G0 X137.4 Y139 Z7 F10000;Dip in**<br>**G4 S1**<br>**G0 X137.4 Y139 Z25 F10000;Dip out**<br>**G0 X137.4 Y139 Z25 F10000**<br>**G0 X137.4 Y139 Z7 F10000;Dip in**<br>**G4 S30**<br>**G0 X137.4 Y139 Z25 F10000;Dip out**<br>**G0 X101.4 Y131 Z25 F10000**<br>**G0 X101.4 Y131 Z7 F10000;Dip in**<br>**G4 S1**<br>**G0 X101.4 Y131 Z25 F10000;Dip out**<br>**G0 X101.4 Y131 Z25 F10000**<br>**G0 X101.4 Y131 Z7 F10000;Dip in**<br>**G4 S1**<br>**G0 X101.4 Y131 Z25 F10000;Dip out**<br>**G0 X101.4 Y131 Z25 F10000**<br>**G0 X101.4 Y131 Z7 F10000;Dip in**<br>**G4 S1**<br>**G0 X101.4 Y131 Z25 F10000;Dip out**	Temperature measurement at well B5; followed by thermocouple cleanup.
**G0 X190 Y148 Z25 F10000;Laser Start Position**<br>**M106 S255**<br>**G4 S60**<br>**M107 S15**<br>**G0 X155.4 Y139 Z25 F10000**<br>**G0 X155.4 Y139 Z7 F10000;Dip in**<br>**G4 S1**<br>**G0 X155.4 Y139 Z25 F10000;Dip out**<br>**G0 X155.4 Y139 Z25 F10000**<br>**G0 X155.4 Y139 Z7 F10000;Dip in**<br>**G4 S1**<br>**G0 X155.4 Y139 Z25 F10000;Dip out**<br>**G0 X155.4 Y139 Z25 F10000**<br>**G0 X155.4 Y139 Z7 F10000;Dip in**<br>**G4 S30**<br>**G0 X155.4 Y139 Z25 F10000;Dip out**<br>**G0 X101.4 Y122 Z25 F10000**<br>**G0 X101.4 Y122 Z7 F10000;Dip in**<br>**G4 S1**<br>**G0 X101.4 Y122 Z25 F10000;Dip out**	Laser irradiation for 1 min and temperature measurement at well B7; followed by thermocouple cleanup.

**G0 X101.4 Y122 Z25 F10000**<br>**G0 X101.4 Y122 Z7 F10000;Dip in**<br>**G4 S1**<br>**G0 X101.4 Y122 Z25 F10000;Dip out**<br>**G0 X101.4 Y122 Z25 F10000**<br>**G0 X101.4 Y122 Z7 F10000;Dip in**<br>**G4 S1**<br>**G0 X101.4 Y122 Z25 F10000;Dip out**	
**G0 X208 Y148 Z25 F10000;Laser Start Position**<br>**M106 S255**<br>**G4 S300**<br>**M107 S15**<br>**G0 X173.4 Y139 Z25 F10000**<br>**G0 X173.4 Y139 Z7 F10000;Dip in**<br>**G4 S1**<br>**G0 X173.4 Y139 Z25 F10000;Dip out**<br>**G0 X173.4 Y139 Z25 F10000**<br>**G0 X173.4 Y139 Z7 F10000;Dip in**<br>**G4 S1**<br>**G0 X173.4 Y139 Z25 F10000;Dip out**<br>**G0 X173.4 Y139 Z25 F10000**<br>**G0 X173.4 Y139 Z7 F10000;Dip in**<br>**G4 S30**<br>**G0 X173.4 Y139 Z25 F10000;Dip out**<br>**G0 X101.4 Y113 Z25 F10000**<br>**G0 X101.4 Y113 Z7 F10000;Dip in**<br>**G4 S1**<br>**G0 X101.4 Y113 Z25 F10000;Dip out**<br>**G0 X101.4 Y113 Z25 F10000**<br>**G0 X101.4 Y113 Z7 F10000;Dip in**<br>**G4 S1**<br>**G0 X101.4 Y113 Z25 F10000;Dip out**<br>**G0 X101.4 Y113 Z25 F10000**<br>**G0 X101.4 Y113 Z7 F10000;Dip in**<br>**G4 S1**<br>**G0 X101.4 Y113 Z25 F10000;Dip out**	Laser irradiation for 5 min and temperature measurement at well B9; followed by thermocouple cleanup.
**G0 X226 Y148 Z25 F10000;Laser Start Position**<br>**M106 S255**<br>**G4 S600**<br>**M107 S15**<br>**G0 X191.4 Y139 Z25 F10000**<br>**G0 X191.4 Y139 Z7 F10000;Dip in**<br>**G4 S1**<br>**G0 X191.4 Y139 Z25 F10000;Dip out**<br>**G0 X191.4 Y139 Z25 F10000**<br>**G0 X191.4 Y139 Z7 F10000;Dip in**	Laser irradiation for 5 min and temperature measurement at well B11; followed by thermocouple cleanup.

**G4 S1<br>G0 X191.4 Y139 Z25 F10000;Dip out<br>G0 X191.4 Y139 Z25 F10000<br>G0 X191.4 Y139 Z7 F10000;Dip in<br>G4 S30<br>G0 X191.4 Y139 Z25 F10000;Dip out<br>G0 X101.4 Y104 Z25 F10000<br>G0 X101.4 Y104 Z7 F10000;Dip in<br>G4 S1<br>G0 X101.4 Y104 Z25 F10000;Dip out<br>G0 X101.4 Y104 Z25 F10000<br>G0 X101.4 Y104 Z7 F10000;Dip in<br>G4 S1<br>G0 X101.4 Y104 Z25 F10000;Dip out<br>G0 X101.4 Y104 Z25 F10000<br>G0 X101.4 Y104 Z7 F10000;Dip in<br>G4 S1<br>G0 X101.4 Y104 Z25 F10000;Dip out**	
**G0 X154 Y130 Z25 F10000;Laser Start Position<br>G4 S30<br>G0 X119.4 Y121 Z25 F10000<br>G0 X119.4 Y121 Z7 F10000;Dip in<br>G4 S1<br>G0 X119.4 Y121 Z25 F10000;Dip out<br>G0 X119.4 Y121 Z25 F10000<br>G0 X119.4 Y121 Z7 F10000;Dip in<br>G4 S1<br>G0 X119.4 Y121 Z25 F10000;Dip out<br>G0 X119.4 Y121 Z25 F10000<br>G0 X119.4 Y121 Z7 F10000;Dip in<br>G4 S30<br>G0 X119.4 Y121 Z25 F10000;Dip out<br>G0 X101.4 Y86 Z25 F10000<br>G0 X101.4 Y86 Z7 F10000;Dip in<br>G4 S1<br>G0 X101.4 Y86 Z25 F10000;Dip out<br>G0 X101.4 Y86 Z25 F10000<br>G0 X101.4 Y86 Z7 F10000;Dip in<br>G4 S1<br>G0 X101.4 Y86 Z25 F10000;Dip out<br>G0 X101.4 Y86 Z25 F10000<br>G0 X101.4 Y86 Z7 F10000;Dip in<br>G4 S1<br>G0 X101.4 Y86 Z25 F10000;Dip out**	Temperature measurement at well D3; followed by thermocouple cleanup.
**G0 X172 Y130 Z25 F10000;Laser Start Position<br>G4 S30**	Temperature measurement at well D5;

**G0 X137.4 Y121 Z25 F10000**<br>**G0 X137.4 Y121 Z7 F10000;Dip in**<br>**G4 S1**<br>**G0 X137.4 Y121 Z25 F10000;Dip out**<br>**G0 X137.4 Y121 Z25 F10000**<br>**G0 X137.4 Y121 Z7 F10000;Dip in**<br>**G4 S1**<br>**G0 X137.4 Y121 Z25 F10000;Dip out**<br>**G0 X137.4 Y121 Z25 F10000**<br>**G0 X137.4 Y121 Z7 F10000;Dip in**<br>**G4 S30**<br>**G0 X137.4 Y121 Z25 F10000;Dip out**<br>**G0 X110.4 Y86 Z25 F10000**<br>**G0 X110.4 Y86 Z7 F10000;Dip in**<br>**G4 S1**<br>**G0 X110.4 Y86 Z25 F10000;Dip out**<br>**G0 X110.4 Y86 Z25 F10000**<br>**G0 X110.4 Y86 Z7 F10000;Dip in**<br>**G4 S1**<br>**G0 X110.4 Y86 Z25 F10000;Dip out**<br>**G0 X110.4 Y86 Z25 F10000**<br>**G0 X110.4 Y86 Z7 F10000;Dip in**<br>**G4 S1**<br>**G0 X110.4 Y86 Z25 F10000;Dip out**	followed by thermocouple cleanup.
**G0 X190 Y130 Z25 F10000;Laser Start Position**<br>**M106 S255**<br>**G4 S60**<br>**M107 S15**<br>**G0 X155.4 Y121 Z25 F10000**<br>**G0 X155.4 Y121 Z7 F10000;Dip in**<br>**G4 S1**<br>**G0 X155.4 Y121 Z25 F10000;Dip out**<br>**G0 X155.4 Y121 Z25 F10000**<br>**G0 X155.4 Y121 Z7 F10000;Dip in**<br>**G4 S1**<br>**G0 X155.4 Y121 Z25 F10000;Dip out**<br>**G0 X155.4 Y121 Z25 F10000**<br>**G0 X155.4 Y121 Z7 F10000;Dip in**<br>**G4 S30**<br>**G0 X155.4 Y121 Z25 F10000;Dip out**<br>**G0 X119.4 Y86 Z25 F10000**<br>**G0 X119.4 Y86 Z7 F10000;Dip in**<br>**G4 S1**<br>**G0 X119.4 Y86 Z25 F10000;Dip out**<br>**G0 X119.4 Y86 Z25 F10000**<br>**G0 X119.4 Y86 Z7 F10000;Dip in**	Laser irradiation for 1 min and temperature measurement at well D7; followed by thermocouple cleanup.

**G4 S1<br>G0 X119.4 Y86 Z25 F10000;Dip out<br>G0 X119.4 Y86 Z25 F10000<br>G0 X119.4 Y86 Z7 F10000;Dip in<br>G4 S1<br>G0 X119.4 Y86 Z25 F10000;Dip out**	
**G0 X208 Y130 Z25 F10000;Laser Start Position<br>M106 S255<br>G4 S300<br>M107 S15<br>G0 X173.4 Y121 Z25 F10000<br>G0 X173.4 Y121 Z7 F10000;Dip in<br>G4 S1<br>G0 X173.4 Y121 Z25 F10000;Dip out<br>G0 X173.4 Y121 Z25 F10000<br>G0 X173.4 Y121 Z7 F10000;Dip in<br>G4 S1<br>G0 X173.4 Y121 Z25 F10000;Dip out<br>G0 X173.4 Y121 Z25 F10000<br>G0 X173.4 Y121 Z7 F10000;Dip in<br>G4 S30<br>G0 X173.4 Y121 Z25 F10000;Dip out<br>G0 X128.4 Y86 Z25 F10000<br>G0 X128.4 Y86 Z7 F10000;Dip in<br>G4 S1<br>G0 X128.4 Y86 Z25 F10000;Dip out<br>G0 X128.4 Y86 Z25 F10000<br>G0 X128.4 Y86 Z7 F10000;Dip in<br>G4 S1<br>G0 X128.4 Y86 Z25 F10000;Dip out<br>G0 X128.4 Y86 Z25 F10000<br>G0 X128.4 Y86 Z7 F10000;Dip in<br>G4 S1<br>G0 X128.4 Y86 Z25 F10000;Dip out**	Laser irradiation for 5 min and temperature measurement at well D9; followed by thermocouple cleanup.
**G0 X226 Y130 Z25 F10000;Laser Start Position<br>M106 S255<br>G4 S600<br>M107 S15<br>G0 X191.4 Y121 Z25 F10000<br>G0 X191.4 Y121 Z7 F10000;Dip in<br>G4 S1<br>G0 X191.4 Y121 Z25 F10000;Dip out<br>G0 X191.4 Y121 Z25 F10000<br>G0 X191.4 Y121 Z7 F10000;Dip in<br>G4 S1<br>G0 X191.4 Y121 Z25 F10000;Dip out**	Laser irradiation for 10 min and temperature measurement at well D11; followed by thermocouple cleanup.

**G0 X191.4 Y121 Z25 F10000<br>G0 X191.4 Y121 Z7 F10000;Dip in<br>G4 S30<br>G0 X191.4 Y121 Z25 F10000;Dip out<br>G0 X137.4 Y86 Z25 F10000<br>G0 X137.4 Y86 Z7 F10000;Dip in<br>G4 S1<br>G0 X137.4 Y86 Z25 F10000;Dip out<br>G0 X137.4 Y86 Z25 F10000<br>G0 X137.4 Y86 Z7 F10000;Dip in<br>G4 S1<br>G0 X137.4 Y86 Z25 F10000;Dip out<br>G0 X137.4 Y86 Z25 F10000<br>G0 X137.4 Y86 Z7 F10000;Dip in<br>G4 S1<br>G0 X137.4 Y86 Z25 F10000;Dip out**	
**G0 X154 Y112 Z25 F10000;Laser Start Position<br>G4 S30<br>G0 X119.4 Y103 Z25 F10000<br>G0 X119.4 Y103 Z7 F10000;Dip in<br>G4 S1<br>G0 X119.4 Y103 Z25 F10000;Dip out<br>G0 X119.4 Y103 Z25 F10000<br>G0 X119.4 Y103 Z7 F10000;Dip in<br>G4 S1<br>G0 X119.4 Y103 Z25 F10000;Dip out<br>G0 X119.4 Y103 Z25 F10000<br>G0 X119.4 Y103 Z7 F10000;Dip in<br>G4 S30<br>G0 X119.4 Y103 Z25 F10000;Dip out<br>G0 X155.4 Y86 Z25 F10000<br>G0 X155.4 Y86 Z7 F10000;Dip in<br>G4 S1<br>G0 X155.4 Y86 Z25 F10000;Dip out<br>G0 X155.4 Y86 Z25 F10000<br>G0 X155.4 Y86 Z7 F10000;Dip in<br>G4 S1<br>G0 X155.4 Y86 Z25 F10000;Dip out<br>G0 X155.4 Y86 Z25 F10000<br>G0 X155.4 Y86 Z7 F10000;Dip in<br>G4 S1<br>G0 X155.4 Y86 Z25 F10000;Dip out**	Temperature measurement at well F3; followed by thermocouple cleanup.
**G0 X172 Y112 Z25 F10000;Laser Start Position<br>G4 S30<br>G0 X137.4 Y103 Z25 F10000<br>G0 X137.4 Y103 Z7 F10000;Dip in**	Temperature measurement at well F5; followed by thermocouple cleanup.

**G4 S1**<br>**G0 X137.4 Y103 Z25 F10000;Dip out**<br>**G0 X137.4 Y103 Z25 F10000**<br>**G0 X137.4 Y103 Z7 F10000;Dip in**<br>**G4 S1**<br>**G0 X137.4 Y103 Z25 F10000;Dip out**<br>**G0 X137.4 Y103 Z25 F10000**<br>**G0 X137.4 Y103 Z7 F10000;Dip in**<br>**G4 S30**<br>**G0 X137.4 Y103 Z25 F10000;Dip out**<br>**G0 X164.4 Y86 Z25 F10000**<br>**G0 X164.4 Y86 Z7 F10000;Dip in**<br>**G4 S1**<br>**G0 X164.4 Y86 Z25 F10000;Dip out**<br>**G0 X164.4 Y86 Z25 F10000**<br>**G0 X164.4 Y86 Z7 F10000;Dip in**<br>**G4 S1**<br>**G0 X164.4 Y86 Z25 F10000;Dip out**<br>**G0 X164.4 Y86 Z25 F10000**<br>**G0 X164.4 Y86 Z7 F10000;Dip in**<br>**G4 S1**<br>**G0 X164.4 Y86 Z25 F10000;Dip out**	
**G0 X190 Y112 Z25 F10000;Laser Start Position**<br>**M106 S255**<br>**G4 S60**<br>**M107 S15**<br>**G0 X155.4 Y103 Z25 F10000**<br>**G0 X155.4 Y103 Z7 F10000;Dip in**<br>**G4 S1**<br>**G0 X155.4 Y103 Z25 F10000;Dip out**<br>**G0 X155.4 Y103 Z25 F10000**<br>**G0 X155.4 Y103 Z7 F10000;Dip in**<br>**G4 S1**<br>**G0 X155.4 Y103 Z25 F10000;Dip out**<br>**G0 X155.4 Y103 Z25 F10000**<br>**G0 X155.4 Y103 Z7 F10000;Dip in**<br>**G4 S30**<br>**G0 X155.4 Y103 Z25 F10000;Dip out**<br>**G0 X173.4 Y86 Z25 F10000**<br>**G0 X173.4 Y86 Z7 F10000;Dip in**<br>**G4 S1**<br>**G0 X173.4 Y86 Z25 F10000;Dip out**<br>**G0 X173.4 Y86 Z25 F10000**<br>**G0 X173.4 Y86 Z7 F10000;Dip in**<br>**G4 S1**<br>**G0 X173.4 Y86 Z25 F10000;Dip out**	Laser irradiation for 1 min and temperature measurement at well F7; followed by thermocouple cleanup.

**G0 X173.4 Y86 Z25 F10000<br>G0 X173.4 Y86 Z7 F10000;Dip in<br>G4 S1<br>G0 X173.4 Y86 Z25 F10000;Dip out**	
**G0 X208 Y112 Z25 F10000;Laser Start Position<br>M106 S255<br>G4 S300<br>M107 S15<br>G0 X173.4 Y103 Z25 F10000<br>G0 X173.4 Y103 Z7 F10000;Dip in<br>G4 S1<br>G0 X173.4 Y103 Z25 F10000;Dip out<br>G0 X173.4 Y103 Z25 F10000<br>G0 X173.4 Y103 Z7 F10000;Dip in<br>G4 S1<br>G0 X173.4 Y103 Z25 F10000;Dip out<br>G0 X173.4 Y103 Z25 F10000<br>G0 X173.4 Y103 Z7 F10000;Dip in<br>G4 S30<br>G0 X173.4 Y103 Z25 F10000;Dip out<br>G0 X182.4 Y86 Z25 F10000<br>G0 X182.4 Y86 Z7 F10000;Dip in<br>G4 S1<br>G0 X182.4 Y86 Z25 F10000;Dip out<br>G0 X182.4 Y86 Z25 F10000<br>G0 X182.4 Y86 Z7 F10000;Dip in<br>G4 S1<br>G0 X182.4 Y86 Z25 F10000;Dip out<br>G0 X182.4 Y86 Z25 F10000<br>G0 X182.4 Y86 Z7 F10000;Dip in<br>G4 S1<br>G0 X182.4 Y86 Z25 F10000;Dip out**	Laser irradiation for 5 min and temperature measurement at well F9; followed by thermocouple cleanup.
**G0 X226 Y112 Z25 F10000;Laser Start Position<br>M106 S255<br>G4 S600<br>M107 S15<br>G0 X191.4 Y103 Z25 F10000<br>G0 X191.4 Y103 Z7 F10000;Dip in<br>G4 S1<br>G0 X191.4 Y103 Z25 F10000;Dip out<br>G0 X191.4 Y103 Z25 F10000<br>G0 X191.4 Y103 Z7 F10000;Dip in<br>G4 S1<br>G0 X191.4 Y103 Z25 F10000;Dip out<br>G0 X191.4 Y103 Z25 F10000<br>G0 X191.4 Y103 Z7 F10000;Dip in**	Laser irradiation for 10 min and temperature measurement at well F11; followed by thermocouple cleanup.

**G4 S30**<br>**G0 X191.4 Y103 Z25 F10000;Dip out**	
**G0 X191.4 Y86 Z25 F10000**<br>**G0 X191.4 Y86 Z7 F10000;Dip in**<br>**G4 S1**<br>**G0 X191.4 Y86 Z25 F10000;Dip out**<br>**G0 X191.4 Y86 Z25 F10000**<br>**G0 X191.4 Y86 Z7 F10000;Dip in**<br>**G4 S1**<br>**G0 X191.4 Y86 Z25 F10000;Dip out**<br>**G0 X191.4 Y86 Z25 F10000**<br>**G0 X191.4 Y86 Z7 F10000;Dip in**<br>**G4 S1**<br>**G0 X191.4 Y86 Z25 F10000;Dip out**<br>**G0 X200.4 Y86 Z25 F10000**<br>**G0 X200.4 Y86 Z7 F10000;Dip in**<br>**G4 S1**<br>**G0 X200.4 Y86 Z25 F10000;Dip out**<br>**G0 X200.4 Y86 Z25 F10000**<br>**G0 X200.4 Y86 Z7 F10000;Dip in**<br>**G4 S1**<br>**G0 X200.4 Y86 Z25 F10000;Dip out**<br>**G0 X200.4 Y86 Z25 F10000**<br>**G0 X200.4 Y86 Z7 F10000;Dip in**<br>**G4 S1**<br>**G0 X200.4 Y86 Z25 F10000;Dip out**	Thermocouple cleanup
**M140 S44; Turn-off bed**<br>**M84 X Y; Disable all steppers but Z**	**End Program**

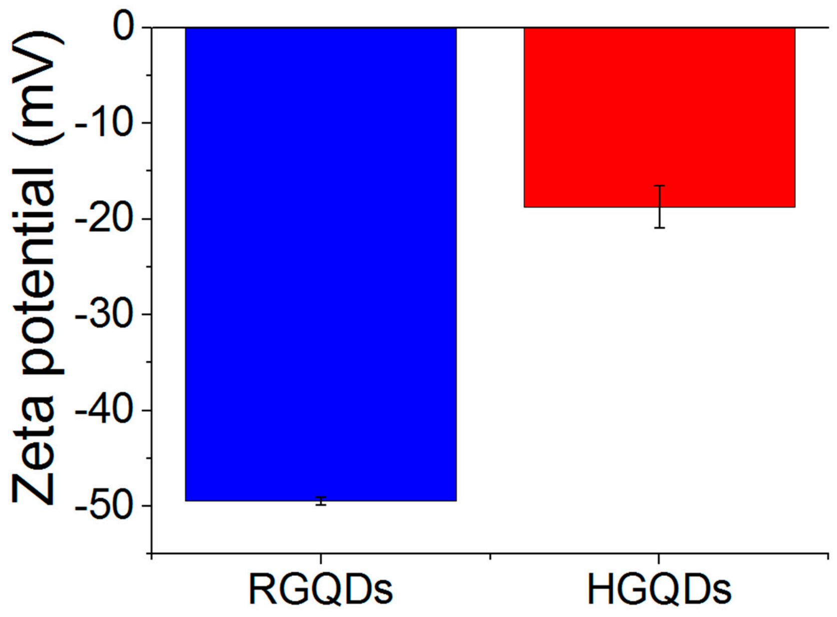

Figure S2. Zeta potential of RGQDs (blue) and HGQDs (red).

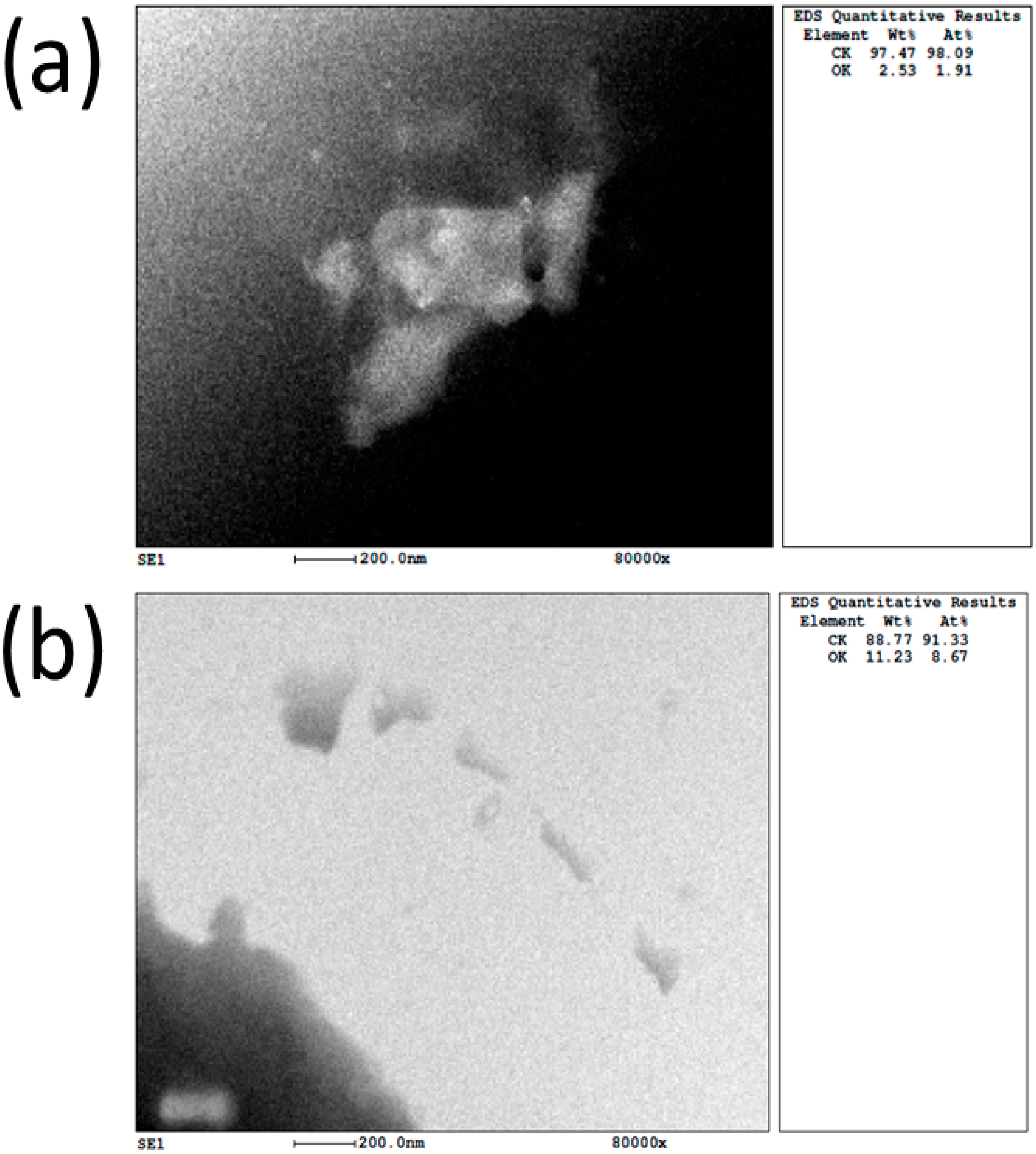

Figure S3. EDX images and atomic percentages of carbon and oxygen for a region with (a) HGQDs and (b) RGQDs.

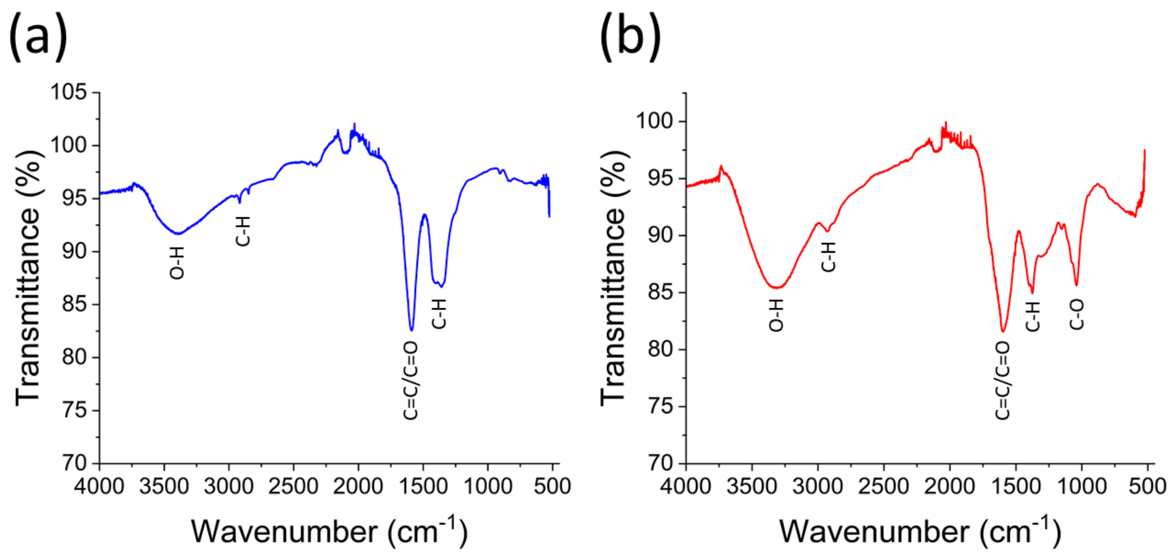

Figure S4. FTIR spectra of (a) RGQDs (blue) and (b) HGQDs (red).

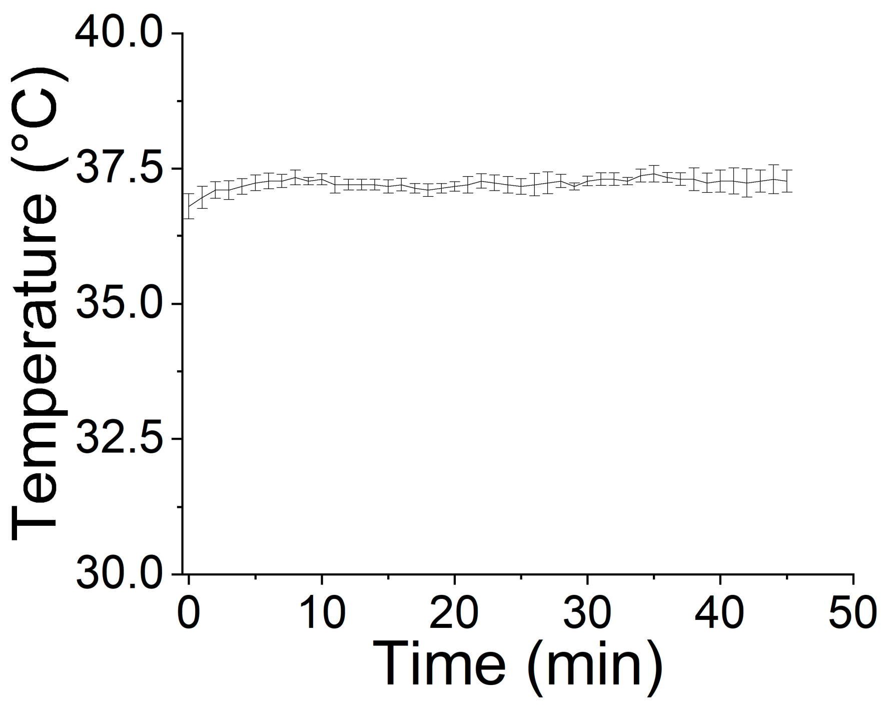

Figure S5. Temperature of water control with 0.9 W/cm^2 808 nm laser irradiation.

Table S2. Tabulated results of Figure 5b.

Well	Irradiation Time (min)	Temperature (°C)	Cell Viability (%)
**RGQDs**			
**Trial 1**			
B3	0	36.9	100
B5	0	36.9	72.7
B7	1	39.4	92.5
B9	5	42.9	32.3
B11	10	43.6	48.6
**Trial 2**			
D3	0	38.2	100
D5	0	37.9	93.1
D7	1	40.1	97.5
D9	5	43.3	49.8
D11	10	43.7	28.6
**Trial 3**			
F3	0	38.4	100
F5	0	38.0	101
F7	1	40.2	92.2
F9	5	43.1	43.4
F11	10	43.3	38.7
**HGQDs**			
**Trial 1**			
B3	0	36.6	100
B5	0	36.7	72.6
B7	1	46.2	39.6
B9	5	55.3	23.5
B11	10	55.1	19.9
**Trial 2**			
D3	0	38.2	100
D5	0	37.8	57.8
D7	1	46.4	62.4
D9	5	55.0	24.1
D11	10	54.4	21.3
**Trial 3**			
F3	0	38.1	100
F5	0	37.8	62.2
F7	1	46.4	38.9
F9	5	54.3	31.0
F11	10	54.1	27.8

Table S3. Table of GQDs developed for PTT applications.

Synthesis Method	Precursor	Temperature (ºC)		Fluorescence		Biocompatibility (mg/mL)	Ref.
		In solution	*In vitro* PTT	NIR	VL		
Bottom-up	Phenol	From 0 to ~18 ºC for 5 min at 0.1 mg/mL with 1064 nm (1.0 W/cm^2)	NA	No	Yes	0.1	1
Bottom-up	Citric acid and urea	From ~24 to 45 ºC for 5 min at 0.8 mg/mL with 808 nm (1.16 W/cm^2)	NA	Yes	Yes	0.5	2
Bottom-up	F. racemosa	From 37 to 45 ºC for 5 min at 0.25 mg/mL with 808 nm (0.5 W/cm^2)	NA	No	Yes	0.25	3
Bottom-up	Hyaluronic acid	From 37 to 46 ºC for 47 min at 1.7 mg/mL with 808 nm (0.9 W/cm^2)	From 37.4 to 54.5 ºC for 10 min at 1.7 mg/mL with 808 nm (0.9 W/cm^2)	Yes	Yes	1.7	Present work
Top-down	RGO	From 37 to 47 ºC for 47 min at 1.5 mg/mL with 808 nm (0.9 W/cm^2)	From 37.6 to 43.5 ºC for 10 min at 1.5 mg/mL with 808 nm (0.9 W/cm^2)	Yes	Yes	1.5	Present work

References

1. Liu, H.; Li, C.; Qian, Y.; Hu, L.; Fang, J.; Tong, W.; Nie, R.; Chen, Q.; Wang, H., Magnetic-induced graphene quantum dots for imaging-guided photothermal therapy in the second near-infrared window. *Biomaterials* **2020,** *232*, 119700.
2. Xuan, Y.; Zhang, R.-Y.; Zhang, X.-S.; An, J.; Cheng, K.; Li, C.; Hou, X.-L.; Zhao, Y.-D., Targeting N-doped graphene quantum dot with high photothermal conversion efficiency for dual-mode imaging and therapy in vitro. *Nanotechnology* **2018,** *29*, 355101.
3. Thakur, M.; Kumawat, M.K.; Srivastava, R. Multifunctional graphene quantum dots for combined photothermal and photodynamic therapy coupled with cancer cell tracking applications. *RSC Adv.* **2017,** *7*, 5251–5261.
